# Clinical evidence for independent regulation of vitamin D by intestinal CYP24A1

**DOI:** 10.1172/JCI190972

**Published:** 2025-04-15

**Authors:** Sandrine Lemoine, Arnaud Molin, Alice Koenig, Justine Bacchetta

**Affiliations:** 1 Service de Néphrologie, Dialyse, Exploration Fonctionnelle Rénale, Hôpital Edouard Herriot, HCL, Lyon, INSERM 1060, France.; 2 Filière ORKID et Filière OSCAR, France.; 3 Université de Caen Normandie, BIOTARGEN UR7450, Service de Génétique, CHU de Caen, Caen, France.; 4 Service de Transplantation, Néphrologie et Immunologie Clinique, Hôpital Edouard Herriot, HCL, Lyon, France.; 5 Service de Néphropédiatrie, Hôpital Femme Mère Enfant, HCL, INSERM 1033, Lyon, France.

**Keywords:** Genetics, Nephrology, Calcium, Chronic kidney disease, Genetic diseases

**To the Editor:** We read with interest the paper from Fuchs et al., showing in mice that intestinal *CYP24A1* can regulate vitamin D locally, independently of systemic regulation by renal CYP24A1 (1). To strengthen the value of these animal data for human health, we report a 61-year-old patient with kidney failure secondary to nephrocalcinosis. Symptoms began at 16 years of age with kidney stone (KS) episodes. Nephrocalcinosis was initially attributed to primary hyperparathyroidism (Figure 1A). Consequently, the patient underwent surgery for primary hyperparathyroidism at 39 years old: two parathyroid glands were removed; only hyperplasia was diagnosed. After surgery, serum calcium increased again (iCa^2+^ 1.48 mmol/L with high parathyroid hormone (PTH) 39 ng/L, *n* = 5.5–38.4), and he subsequently received cinacalcet to treat hypercalcemia.

PTH was only moderately increased in the setting of chronic kidney disease (CKD) (203 μmol/L creatinine, 35 mL/min/1.73 m^2^ estimated glomerular filtration rate [eGFR]). No 1,25-(OH)_2_vitamin D was measured at the time, but sarcoidosis was ruled out (PET-CT and angiotensin-converting enzyme were negative). Hypercalcemia recurred after a second surgery of the third parathyroid gland. Dialysis was initiated at 57 years of age. The patient underwent kidney transplantation from an human leukocyte antigen–identical (HLA-identical) living donor (his brother) two years later (59 years old). The patient remained hypercalcemic with a nonadapted PTH, explaining why he remained under cinacalcet after transplantation.

Seven months after transplant, the patient experienced an episode of obstructive pyelonephritis (a 9 mm graft stone identified on CT scan [Figure 1B]). Since the patient was transplanted with his brother’s kidney, without any KS on the predonation evaluation, this finding suggested a de novo stone formation. Following this episode, a genetic analysis was performed, revealing a homozygous recurrent loss-of-function *CYP24A1* pathogenic variant NM_000782.5:c.1186C>T (p.Arg396Trp), rs114368325 (2). There is no specific evidence supporting the pathogenicity of this variant in a heterozygous state, but numerous patient observations and animal studies strongly support its pathogenicity in a homozygous or compound heterozygous state (3). His brother did not carry this variant, either in a heterozygous or homozygous state, and was in good health without KS. Figure 1C describes the timeline of medical history.

A complete blood work and 24-hour urinary collection were performed: it showed normal serum calcium (2.39 mmol/L), 49 ng/L (*n* = 15–65) PTH under cinacalcet, 32 ng/mL 25-OH vitamin D, 100 pmol/L 1,25-(OH)_2_vitamin D, 171 μmol/L creatinine, 37 mL/min/1.73 m² eGFR, 2.80 mmol/24 hours calciuria, 0.20 mmol/24 hours citraturia, and 300 μmol/24 hours oxaluria. Normal calciuria and reduced citrate levels are explained by advanced CKD. Levels of 1,25-(OH)_2_vitamin D were elevated for a CKD patient, without hypercalcemia, likely due to a counterbalance between CKD and the *CYP24A1* defect. Although cinacalcet has been discussed as inducing nephrocalcinosis after transplantation (4), stone recurrence may not be attributed to the treatment, since PTH always remained normal after transplantation. As we ruled out additional causes of stones, we suggest that intestinal deregulation of vitamin D metabolism due to *CYP24A1* loss-of-function variant, uncompensated by the grafted kidney *CYP24A1* enzyme, may increase calcium intestinal absorption as described in *CYP24A1* intestinal conditional knockout mice fed a high-calcium diet by Fuchs et al., leading to recurrent KS.

Thus, this case report highlights that patients with CYP24A1 defect (a) can be challenging to diagnose, especially in CKD with secondary/tertiary hyperparathyroidism counterbalancing the effects of *CYP24A1* variants, and (b) can display nephrolithiasis recurrence even after graft with a kidney with intact CYP24A1 function. This recurrence underscores the hypothesis that intestinal CYP24A1 may play a role in phosphate/calcium metabolism. While reinforcing Fuchs’ data in mice, nephrologists should keep in mind that KS can recur after transplantation and genetic testing should be done whenever nephrocalcinosis occurs with CKD.

## Figures and Tables

**Figure 1:**
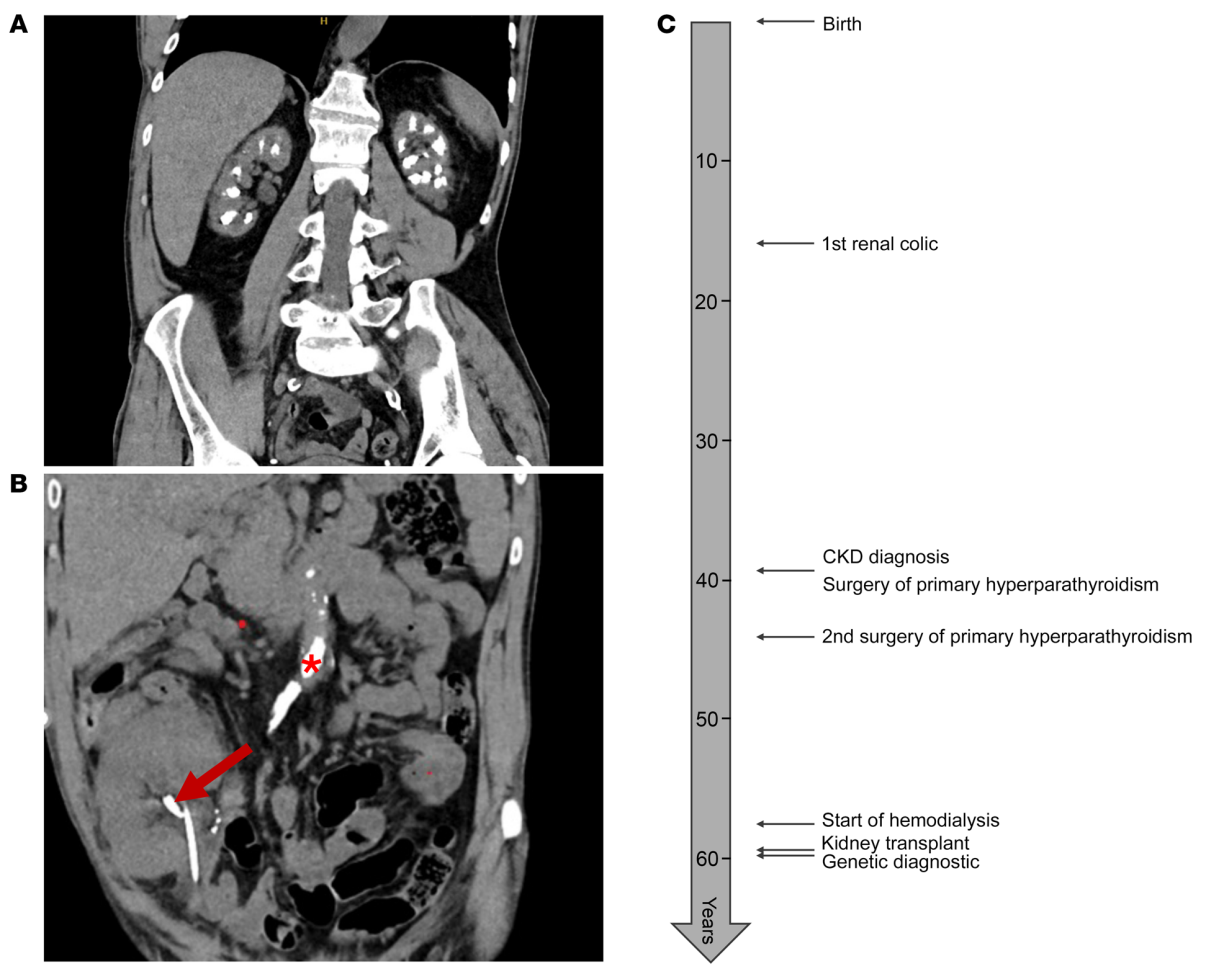
Kidney imaging and timeline of medical history. (**A**) Native kidney with nephrocalcinosis. (**B**) Kidney transplant with ureteral stent and KS (9 mm) shown by the red arrow. Aortic calcification (red star). (**C**) Timeline describing the patient’s medical history

## Version 1 04/15/2025

Electronic publication
